# Outcome domains measured in randomized controlled trials of physical activity for older adults: a rapid review

**DOI:** 10.1186/s12966-023-01431-3

**Published:** 2023-03-24

**Authors:** Dawn C. Mackey, Christina L. Ekegren, Claire Baldwin, Peter J. Young, Samantha M. Gray, Alex Ciok, Angela Wong

**Affiliations:** 1 Department of Biomedical Physiology and Kinesiology, Simon Fraser University, 8888 University Drive, Burnaby, British Columbia V5A 1S6 Canada; 2 Rehabilitation, Ageing and Independent Living (RAIL) Research Centre, School of Primary and Allied Health Care, Monash University, Peninsula Campus, Building G, Moorooduc Hwy, Frankston, Victoria 3199 Australia; 3 Caring Futures Institute, College of Nursing and Health Sciences, Flinders University, Level 1, Room N103, Sturt North Sturt Rd, Bedford Park, South Australia 5042 Australia

**Keywords:** Knowledge synthesis, Core outcome set, Minimum data set, Standardized outcomes, Seniors, Exercise

## Abstract

**Background:**

Toward development of a core outcome set for randomized controlled trials (RCTs) of physical activity (PA) interventions for older adults, the purpose of this study was to identify outcome domains and subdomains (‘what’ was measured) in previously published RCTs of PA for older adults.

**Methods:**

We conducted a rapid review and searched Ovid MEDLINE for recently- published (2015-2021), English-language, RCTs of PA interventions for older adults (mean age 60+ yrs). We limited to articles published in Web of Science top-10 journals in general and internal medicine, geriatrics and gerontology, rehabilitation, and sports science. Two reviewers independently completed eligibility screening; two other reviewers abstracted trial descriptors and study outcomes. We classified study outcomes according to the standard outcome classification taxonomy endorsed by the Core Outcome Measures in Effectiveness Trials Initiative.

**Results:**

Our search yielded 548 articles; 67 articles were eligible to be included. Of these, 82% were efficacy/effectiveness trials, 85% included both male and female participants, and 84% recruited community-dwelling older adults. Forty percent of articles reported on interventions that involved a combination of group and individual PAs, and 60% involved a combination of PA modes (e.g., aerobic, resistance). Trial sample size ranged from 14 to 2157 participants, with median (IQR) of 94 (57-517); 28,649 participants were included across all trials. We identified 21 unique outcome domains, spanning 4/5 possible core areas (physiological/clinical; life impact; resource use; adverse events). The five most commonly reported outcome domains were physical functioning (included in *n*=51 articles), musculoskeletal and connective tissue (*n*=30), general (*n*=26), cognitive functioning (*n*=16), and emotional functioning/wellbeing (*n*=14). Under these five outcome domains, we further identified 10 unique outcome subdomains (e.g., fall-related; body composition; quality of life). No outcome domains or subdomains were reported consistently in all RCTs.

**Conclusions:**

We found extensive variability in outcome domains and subdomains used in RCTs of PA for older adults, reflecting the broad range of potential health benefits derived from PA and also investigator interest to monitor a range of safety parameters related to adverse events. This study will inform development of a core outcome set to improve outcome reporting consistency and evidence quality.

**Supplementary Information:**

The online version contains supplementary material available at 10.1186/s12966-023-01431-3.

## Background

Physical activity (PA) is a strongly recommended intervention for older adults, as randomized controlled trials (RCTs) have demonstrated positive effects on a variety of outcome domains (e.g., falls, cognition, mobility, and mood, among others) [1–4]. However, PA trialists lack appropriate guidance on which outcome domains to measure consistently, leading to considerable heterogeneity in outcome selection and reporting across PA RCTs for older adults. This heterogeneity contributes to bias and makes it difficult to compare, contrast, and combine results across trials [5–7]. In turn, lack of consistency in outcome selection and reporting makes it challenging to identify effective, ineffective, and unproven PA interventions in a timely manner, and thereby impedes future health research, health care decision making, and development of PA policy and public health programs to support aging.

For example, given strong associations with all-cause mortality [8] as well increased dependence in activities of daily living, hospitalization, and entry into nursing homes [9, 10], slow gait speed has been called a vital sign for older adults, and leading scholars and geriatricians have proposed that a gait speed of 0.6 m/s or slower should be considered as a diagnosis of dismobility [11]. However, gait speed is not measured consistently across PA RCTs for older adults, which impairs our ability to identify and implement the most effective interventions for increasing gait speed.

Defining a minimum and standard set of outcome domains to measure and report in all PA RCTs for older adults – called a core outcome set (COS) – would help to address the aforementioned problems [7]. Development and implementation of a COS leads to higher-quality evidence about interventions and less research waste [7, 12]. In turn, interventions that work may be available more quickly to those who need them, for example through scaling up of effective PA interventions to reach large populations [13–15].

The Core Outcome Measures in Effectiveness Trials (COMET) Initiative, launched in 2010, fosters the development, application, and promotion of COS in all health areas and supports collaboration among those developing COS [16]. Use of COS are endorsed by the Consolidated Standards of Reporting Trials 2010 statement [17] and the Standard Protocol Items: Recommendations for Interventional Trials 2013 statement [18, 19]. The field of COS was pioneered by the Outcome Measures in Rheumatology consensus initiative, which began in 1992 and has developed COS for many rheumatologic conditions, including osteoarthritis [20]. A COS for RCTs in the area of fall and injury prevention [21] has seen good adoption [22] and thereby enabled the production of high-quality systematic reviews and meta-analyses that summarize evidence in the field (e.g., [2]).

A critical first step in COS development is to review existing literature to determine which outcomes domains (and subdomains) have been measured and reported in past trials [23]. Accordingly, to support future development of a COS for RCTs of PA for older adults, the aim of this study was to conduct a rapid review to identify outcome domains and subdomains that have been used in RCTs of PA for older adults.

## Methods

### Protocol

We developed and followed a rapid review protocol using guidance from existing literature [24–28]. A checklist for reporting rapid reviews does not currently exist in the Enhancing the QUAlity and Transparency Of health Research library, but this study is reported according to the relevant quality elements of the Preferred Reporting Items for Systematic Reviews and Meta-Analyses (PRISMA) extension for scoping review (PRISMA-ScR) [29], to which the current study most closely aligns.

### Inclusion criteria

Consistent with rapid review methodology, which streamlines components of the systematic review process, we sought to include recently published, English-language, high-quality RCTs that delivered a PA intervention to older adults with the goal of affecting one or more health-related outcomes. Operational definitions of each of these concepts are included in Table 1.

**Table 1:** Key concepts and operational definitions pertaining to the rapid review

**Concept**	**Operational Definition**
Recently published	2015-current (Feb 2021)
High-quality	Journal with a top-10 Web of Science impact factor in the following four categories: Medicine, General & Internal; Geriatrics and Gerontology; Rehabilitation; and Sports Science.
Randomized controlled trials	An experiment in which two or more interventions, possibly including a control intervention or no intervention, are compared by being randomly allocated to participants. In most trials one intervention is assigned to each individual but sometimes assignment is to defined groups of individuals (for example, in a household) or interventions are assigned within individuals (for example, in different orders or to different parts of the body) [30].
Physical activity intervention	Physical activity is any bodily movement produced by skeletal muscles that results in energy expenditure and increases heart rate and breathing. Exercise is physical activity that is planned, structured, repetitive and purposive in the sense that improvement or maintenance of one or more components of physical fitness is an objective [31]. The following types of interventions were eligible: • Trials must have delivered some type of physical activity to some participants with the goal of affecting one or more health-related outcomes (other than just physical activity behaviour). • In multicomponent interventions, physical activity must have been one of the interventions delivered. • In multifactorial interventions, where interventions were targeted to individual risk factors, physical activity must have been one of the interventions available. • In factorial designs that tested physical activity with one or more other active interventions, at least one group had to receive physical activity alone (possibly with control), so that the effects of physical activity could be isolated. The following types of interventions were not eligible: • Behaviour change interventions whose main purpose was to increase levels of physical activity. • Interventions that delivered the same physical activity to all participants and augmented it with another intervention (e.g., dietary). • Single joint rehabilitation interventions.
Older adults	Mean or median age of study population at least 60 years [32]. We did not exclude trials based on living arrangements of older adults; thus, trials were eligible if they focused on older adults living in the community, assisted living, and residential care/aged-care/nursing homes/long-term care homes. We excluded trials where the intervention was conducted in a hospital setting or on a hospital-based population, as hospital stays are temporary, and usually represent a fluctuating health status and a unique set of barriers, enablers, and goals for physical activity [33]. We excluded trials that targeted and recruited a specific clinical population (e.g., all participants had obesity/overweight, cardiovascular disease, diabetes, osteoarthritis, osteoporosis, frailty); however, we included trials where some participants had one or more clinical conditions. In addition, we did not regard people with a history of falls as a specific clinical population because falling is a highly-prevalent behaviour among older adults.

### Search strategy

We searched MEDLINE [Ovid], since all journal titles of interest were indexed in MEDLINE. We searched first for journal titles of interest. Next, we used a combination of MESH terms and keywords to search the concepts of PA and older adult. We used the MEDLINE [Ovid] filter for RCTs. We limited by year of publication (2015 to 2021), and English language. An information scientist reviewed and provided feedback on the search strategy to optimize sensitivity and specificity. The search strategy is included in Additional file 1.

### Screening process

We imported and managed articles in Covidence software for screening. Two reviewers (DCM, CLE) independently screened articles against eligibility criteria in two phases: citations (title and abstract screening); and full-text article screening. We resolved discrepancies by discussion.

### Data abstraction process

We defined the data items for abstraction a priori, which included the following main categories: article information (e.g., first author, year of publication, journal title), study characteristics (e.g., country, continent, phase of trial, clinical trial registration), study design (e.g., study setting, sample size, % female participants, mean participant age, study arms, randomization unit), interventions (e.g., primary location, mode, type), adverse events, intervention adherence, and study outcomes.

We imported and managed articles in NVivo software for data abstraction. Data were abstracted from articles by highlighting the relevant content, assigning it to its corresponding node (data item), and exporting tables of highlighted content. In addition, categorical data items (e.g., was clinical trial registered: yes, no; participant sex: females only, males only, female and male sexes) were classified directly on a Google spreadsheet with pre-defined data validation.

Two reviewers (AC, AW) independently abstracted the study outcomes from all articles and resolved discrepancies through discussion. A third reviewer (DCM) verified the set of outcomes abstracted from all articles. All other data items were abstracted by a single reviewer (AC or AW). Reviewers completed their abstraction for each item from all articles before beginning abstraction of the next item, and they sought clarification and assistance when needed from each other and from DCM.

### Synthesis

We classified study outcome domains according to the standard outcome classification taxonomy endorsed by the COMET initiative, which includes five core areas (death, physiological/clinical, life impact, resource use, and adverse events) and 38 outcome domains [23]. To increase descriptive power, we also defined custom outcome *subdomains* for those domains that appeared in at least 10 articles, as we felt that outcome representation across approximately 15% of included studies would give sufficient breath to understand ‘what’ may be critically important to measure in trials of PA in older adults. This is consistent with the objectives of this review to classify outcome domains and subdomains, which constitute ‘what’ to measure, rather than psychometric properties of outcome instruments, which constitute ‘how’ to measure. The core areas, outcome domains, and outcome subdomains we applied and used for results presentation are listed in Additional file 2. Applying the COMET taxonomy to some outcomes required discussion among the researchers; decisions about outcome classification are detailed in Additional file 3. Notably, some outcomes were classified under multiple domains, and specifically named adverse events (e.g., musculoskeletal injury) were categorized under the appropriate taxonomy domain and not under the generic adverse event domain [23].

## Results

### Literature search & screening

**Fig. 1:**
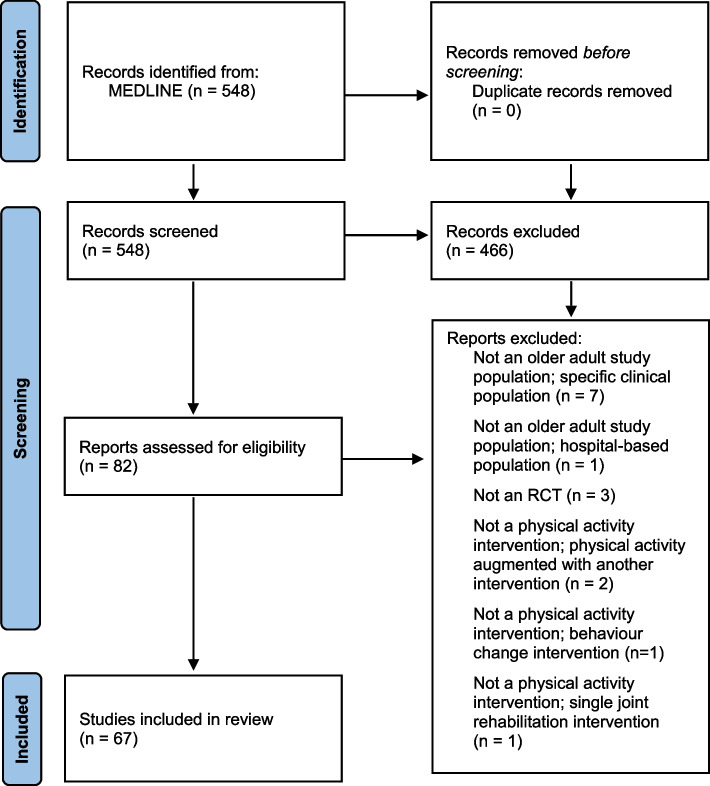
PRISMA flow diagram for rapid review

Our search yielded 548 citations (titles and abstracts). Citations were screened in four batches (*n*=25, *n*=25, *n*=150, *n*=348). Among the 548 citations screened, both reviewers (DCM, CLE) agreed on eligibility status for 514 (93.8%) and disagreed on 34 (6.2%), Cohen’s Kappa = 0.77. Reviewers readily resolved discrepancies by email or videoconference and made minor updates to the wording of eligibility criteria to improve clarity. In total, 82/548 (15.0%) articles passed citation screening and moved onto full text screening (Fig. 1. PRISMA flow diagram for rapid review) [34]. During full text screening of 82 articles, there were 13 discrepancies (15.9%); 12 disagreements about inclusion/exclusion (Cohen’s Kappa = 0.45), and one disagreement about reason for exclusion. These were readily resolved by email correspondence and brief discussion. Of the 67 articles that passed full text screening and were included in the review (Additional file 4), 30 were published in Geriatrics and Gerontology journals, 15 in Rehabilitation journals, 13 in Sports Science journals, and 9 in Medicine, General & Internal journals.

### Descriptive characteristics of randomized controlled trials

Of the 67 included articles, 35 (52%) were from trials in North America, 14 (21%) from Europe, eight (12%) from Asia, seven (10%) from Oceania (all in Australia), and three (5%) from South America (Table 2). In North America, 33 articles were from the United States and two from Canada. Clinical trial registration was reported in 41 (61%) articles; the most common registries were ClinicalTrials.gov (*n*=29) and the Australian New Zealand Clinical Trials Registry (*n*=8).

**Table 2:** Descriptive characteristics of included trials (*n*=67)

**Characteristic**	**n (%)**	**Characteristic**	**n (%)**
Continent where trial was conducted		Publication stage	
North America	35 (52)	M﻿ain results	49 (73)
South America	3 (5)	S﻿econdary analysis/results	11 (16)
Europe	14(21)	S﻿ubgroup analysis	5 (8)
Asia	8 (12)	P﻿rotocol	1 (1.5)
Oceania	7 (10)	Other	1 (1.5)
Country where trial was conducted		Phase of trial	
United States	32 (48)	E﻿fficacy/effectiveness	55 (82)
Australia	7 (10)	P﻿ragmatic	1 (1)
Finland	4 (6)	F﻿easibility/pilot	10 (15)
Japan	4 (6)	O﻿ther	1 (1)
Other	24 (36)		
Clinical trial registry		Group design	
ClinicalTrials.gov	29 (43)	P﻿arallel	63 (94)
ANZCTR	8 (12)	C﻿ross-Over	0 (0)
ISRCTN	2 (3)	F﻿actorial	4 (6)
Other	2 (3)	A﻿daptive	0 (0)
Not registered	26 (39)	S﻿tepped-wedge	0 (0)
Sample size calculation		Randomization	
Yes	28 (41)	I﻿ndividual	61 (91)
No	39 (59)	C﻿luster	6 (9)
Study setting		Type of control group	
Community	56 (84)	A﻿ctive	36 (54)
Assisted living/low-level care	1 (1)	W﻿aitlist	2 (3)
Long-term care/ high-level care	4 (6)	P﻿lacebo/usual care	24 (36)
Other	6 (9)	N﻿ot identified	5 (7)
Participant sex		PA intervention, primary type	
Males and females	57 (85)	C﻿ombination	40 (60)
Females only	10 (15)	F﻿lexibility	0 (0)
Males only	0 (0)	A﻿erobic/cardiorespiratory	9 (13)
Study goal		S﻿trength/resistance	13 (19)
Superiority	67 (100)	B﻿alance/proprioceptive	5 (7)
Noninferiority/equivalence	0 (0)		
Intervention combination		Intervention adherence measured?	
Single	52 (78)	Y﻿es	52 (78)
Multicomponent	14 (21)	N﻿o	15 (22)
Multifactorial	1 (1)	Intervention adherence reported?	
PA intervention mode		Y﻿es	48 (72)
Group	19 (28)	N﻿o	19 (28)
Combination	27 (40)	Adverse events reported?	
Individual	13 (19)	Y﻿es	45 (67)
Unspecified	8 (12)	N﻿o	22 (33)

*Note*: *ANZCTR* Australian New Zealand Clinical Trials Registry, *ISRCTN* International Standard Randomized Controlled Trial Number, *PA* Physical Activity

Individual trial sample size ranged from 14 to 2157 participants, with median (IQR) of 94 (57-517); a total of 28,649 participants were included across all trials. A sample size calculation was included in 28 (41%) articles (Table 2). Trials were most commonly conducted in community settings (*n*=56, 84%), but four (6%) were conducted in nursing homes, one in assisted living, and six (9%) in other settings (e.g., research facility). Males and females were recruited in 57 (85%) trials; 10 (15%) trials recruited females only, and no trials recruited males only. Study populations were predominantly female (% female ranged from 40 to 100% with mean (SD) of 72 (15)).

Of the 67 included articles, 49 (73%) reported on main trial results, while 11 (16%) reported a secondary analysis, and five (8%) a subgroup analysis (Table 2). One article reported a trial protocol and one other article reported an ancillary study from a larger trial. Fourteen (21%) articles reported on data from either the Lifestyle Interventions and Independence for Elders (LIFE) Study [1] or the LIFE Pilot Study [35].

All 67 trials were superiority trials (designed to assess if one or more interventions was *different* from – better or worse – than control) [36] (Table 2). The majority were designed to test intervention efficacy/effectiveness (*n*=55, 82%), while 10 (15%) were proof-of-concept/feasibility/pilot trials, one was a large pragmatic trial, and one a cost-effectiveness trial. Sixty-three (94%) trials used a parallel study design, while four (6%) used a factorial design; no trials used cross-over, adaptive, or stepped wedge designs. Individual randomization was used in 61 (91%) trials and cluster randomization in six (9%).

Forty-two (63%) trials had two arms, 18 (27%) had three arms, and seven (10%) had 4+ arms. Active control groups were used in 36 (54%) trials, placebo/usual care control groups in 24 (36%), and waitlist control groups in two (3%) (Table 2); a control group was not explicitly identified in five (7%) trials. Most intervention arms used a combination of PA types, while 13 (19%) trials focused solely on strength/resistance activities, nine (13%) on aerobic/cardiorespiratory, and five (7%) on balance/proprioception. Many trials (*n*=27, 40%) also used a combination of group and individual modes of PA, while 19 (28%) were comprised solely of group-based activities, 13 (19%) were solely individual activities; mode was not reported in eight (12%) trials. Intervention adherence was reported to have been measured in 52 (78%) trials; however, measured intervention adherence was reported in only 48 (72%) trials. Adverse events were reported on in 45 (67%) trials.

### Classification of study outcomes into domains and subdomains

We identified 21 of 38 possible outcome domains across the 67 articles, spanning 4 of 5 possible core areas: physiological/clinical, life impact, resource use, and adverse events (Table 3). No outcome domains were identified under the core area of death. Further, no outcome domains were reported in all RCTs.

**Table 3:** Outcome frequencies and examples (classified under core area; outcome domain; outcome subdomain) in *n*=67 articles

Outcome domain	Articles with domain reported (n)	Outcome subdomain (if applicable)	Articles with subdomain reported (n)	Example outcomes
***Core Area = Physiological/Clinical***				
Musculoskeletal and connective tissue outcomes	30			
		Muscle performance	23	Muscle strength, muscle power, muscle torque, muscle quality, muscle fatigue
		Bone	9	Fractures, bone mineral density, bone mineral content, bone strength, bone area
		Body composition	8	Muscle mass, muscle area, muscle density, lean mass, fat mass, body fat %, visceral adipose tissue, intramuscular fat area
		Other	2	Muscle fiber type assessments, muscle protein synthesis, gene expression (e.g., mTOR, FOXO1, MSTN)
General outcomes	26			
		Fall-related	15	Number of falls, cumulative fall incidence, fall rates, number of fallers, proportion of fallers, risk factors for falls (e.g., Physiological Profile Assessment)
		Body composition	6	Body weight, body mass index, waist circumference, hip circumference, waist-to-hip ratio
		Other	8	Balance, postural sway, frailty, components of frailty phenotype (e.g., exhaustion, involuntary weight loss), new diagnoses of chronic disease, quality of well-being
Injury and poisoning outcomes	8			Fractures, fall-induced fractures, injurious falls, rate of injurious falls, injured fallers, microbleeds
Cardiac outcomes	7			Cardiorespiratory fitness (e.g., VO2 peak), blood pressure, pulse pressure, cholesterol, triglycerides, cardiac output, heart rate, apo B-48
Endocrine outcomes	5			IGF-1, DHEA-S, PTH, glucose, resistin
Eye outcomes	1			Contrast sensitivity
Immune system outcomes	5			CRP, IL-15, IL-8, IL-6, IL-10, IL-1B, IL-RA, Bad, Bax, Bcl-1, Bcl-xL, Bcl-2, GABARAP, TLR-1, RelA, NFKB1, caspase-1, pro-caspase-1, caspase-3, NLRP3, LAMP-2, beclin-1, Ser757, phospho-ULK-1, Atg16, Atg12, p62/SQSTMI, LC3II, LC3I, GM-CSF, CD8+/-CD28+/-CD57+/-
Infection and infestation outcomes	1			Number of infections over time
Metabolism and nutrition outcomes	4			TRAP, AOPP, FOX, NOx, regional cerebral FDG uptake, 25(OH)D
Nervous system outcomes	6			Proprioception, reaction time, contraction velocity, muscle activation, white matter hyperintensities, BDNF, incident mild cognitive impairment or dementia, brain functional connectivity
Psychiatric outcomes	2			Depression, incident mild cognitive impairment or dementia
Respiratory and thoracic and mediastinal outcomes	7			VO2 peak, VO2 max, inspiratory capacities, expiratory capacities, volumes (e.g., chest well, pulmonary rib cage)
Vascular outcomes	4			Diastolic blood pressures, systolic blood pressures, total cholesterol, triglycerides, high-density lipoprotein cholesterol, low-density lipoprotein cholesterol, protein apolipoprotein B-48, Maximal a-vO2diff, carotid to femoral pulse wave velocity, carotid artery compliance, carotid diameter, carotid IMT diameter, carotid IMT/lumen diameter, tortuosity ratio, straight venous length, tortuous venous length, microbleeds, vascular endothelial growth factors
***Core Area = Life Impact***				
Physical functioning	51			
		Mobility	44	Physical performance (e.g., Short Physical Performance Battery, Timed Up and Go, gait speed, sit-to-stand tests), gait variability and smoothness, mobility disability, life-space
		Fall-related	16	Number of falls, cumulative fall incidence, fall rates, number of fallers, proportion of fallers, risk factors for falls
		Lifestyle	16	Physical activity behaviour (objective and self-report measures), sedentary behaviour, diet questionnaires, caloric intake
		Balance	10	Functional reach, standing balance tests including single leg stance
		Quality of life	10	Quality of life assessments (e.g., SF-36, SF-12, SF-8, EQ-5D-5L, Quality of Well-Being Scale)
		Other	5	Back scratch test, sit-and-reach tests, time to exhaustion on maximal exercise tests
Social functioning	8			Quality of life assessments (e.g., SF-36, SF-12, SF-8, EQ-5D-5L, Quality of Well-Being Scale)
Role functioning	7			Quality of life assessments (e.g., SF-36, SF-12, SF-8, EQ-5D-5L, Quality of Well-Being Scale)
Emotional functioning/wellbeing	14			
		Fall-related	9	Falls efficacy, fear of falling, balance confidence
		Quality of life	9	Quality of life assessments (e.g., SF-36, SF-12, SF-8, EQ-5D-5L, Quality of Well-Being Scale)
Cognitive functioning	16			
		Multiple cognitive functions	10	Montreal Cognitive Assessment, Modified MiniMental State Exam, Wechsler Adult Intelligence Scale-III Digit Symbol Coding test, Addenbrooke’s Cognitive Evaluation revised
		Processing speed & executive functioning	8	Trail Making Tests A and B, Digit Symbol Substitution Test, n-back task, Flanker Inhibitory Control and Attention Test, task-switching paradigm tests, verbal digit span forward and backward test, Stroop Color-Word Test
		Other	5	Hopkins Verbal Learning Test-Revised, Rey Auditory Verbal Learning Test, choice step reaction time
Delivery of care	3			Enjoyment, perceived tolerance, intervention satisfaction, intervention adherence
***Core Area = Resource Use***				
Economic	3			Quality adjusted life years, intervention costs, health care utilization, health and community and social service use, health care costs, incremental cost-effectiveness ratio
***Core Area = Adverse Events***				
Adverse events/effects	1			Occurrence of adverse events

The five most commonly reported outcome domains were physical functioning (included in *n*=51 articles), musculoskeletal and connective tissue outcomes (*n*=30), general outcomes (*n*=26), cognitive functioning (*n*=16), and emotional functioning/wellbeing (*n*=14) (Table 3). These five outcome domains were also the ones that met our criteria for defining subdomains, as they each appeared in at least 10 articles (Table 3). General outcomes referred to disorders and global measures and symptoms that affected the whole body and could not be attributed to a specific bodily system (see example outcomes in Table 3) [23].

We identified 10 unique outcome subdomains. For the physical functioning domain, we defined six subdomains (in order of frequency): mobility, fall-related, lifestyle, balance, quality of life, and other. For the musculoskeletal and connective tissue outcomes domain, we defined four subdomains: muscle performance, bone, body composition, and other. For the general outcomes domain, we defined three subdomains: fall-related, body composition, and other. For the cognitive functioning domain, we defined three subdomains: multiple cognitive functions, processing speed & executive function, and other. Finally, for the emotional functioning/wellbeing domain, we defined two subdomains: fall-related and quality of life. Notably, some subdomains were common to multiple domains, such as fall-related, body composition, and quality of life. We grouped outcomes found in *n*=2 articles or fewer into a category called “other.” Examples of outcomes classified under each domain, and subdomain (where applicable) are given in Table 3.

## Discussion

In this rapid review, we observed considerable heterogeneity in outcome domains measured and reported in recently published, high-quality RCTs of PA for older adults, predominantly conducted in the community setting. Twenty-one unique outcome domains and 10 unique outcome subdomains were reported across 67 articles. This finding reflects the broad range of health benefits potentially derived from PA and also investigator interest to monitor a range of safety parameters that may be related to adverse events from PA. Notably, no outcome domains or subdomains were reported in all trials, which underscores the need to develop a COS to improve outcome reporting consistency and enhance evidence quality.

Physical functioning was the most dominant outcome domain reported in the RCTs included in this review, indicating that PA trialists have traditionally emphasized the importance of measuring the physical functioning benefits from PA. The physical functioning domain encompassed a number of distinct subdomains, the most prominent of which was mobility. The measures of mobility included in the articles we reviewed largely captured an individual’s capacity for mobility by assessing physical performance, such as gait speed, chair stand performance, etc. There were not many instances where measures of a person’s enacted mobility, such as the extent and frequency of movement away from one’s residence (which may further relate to role or social functioning), were used as outcomes. As a result, there is opportunity for more holistic and comprehensive measurement of mobility in future PA RCTs [37, 38].

Musculoskeletal and connective tissue was the second most dominant outcome domain in this review. Within, the most prominent subdomain was muscle performance, which included measures of muscle strength, power, torque, quality, and fatigue. This emphasis on muscle performance was likely due to the nature of interventions in the included trials, whereby about 20% exclusively targeted strength/resistance training and another 60% included combination training, some of which incorporated strength/resistance exercises. Strength/resistance training is heavily promoted for older adults for the prevention of falls and related injuries [2].

Fall-related outcomes also appeared prominently in the articles reviewed. The subdomain of fall-related outcomes was associated with three different outcome domains – physical functioning, general, and emotional – reflecting the wide range of impacts that falls have on the daily lives of older adults and the potential of PA to mitigate or prevent these impacts.

None of the trials included in this review measured the impacts of PA on sleep. As the importance of good sleep quality and quantity to overall health and wellbeing continue to be explored and documented [39, 40], we hypothesize that older adults and their health care providers will deem positive impacts on sleep to be an important reason for participation in PA. In addition, under the core area of resource use, the outcome domains of ‘hospital’, ‘need for further intervention’, and ‘society/carer burden’ were not reported in included articles. This may represent (1) a lack of input from health care professionals and/or older adult consumers in trial outcome selection, (2) a lack of suitable outcome measures (in terms of measurement feasibility, data accessibility, or psychometric performance), (3) limitations in trial resources (e.g., sample size required) and/or (4) a lack of expertise in health economics among teams of trial investigators. Future research will be necessary to determine the outcomes related to PA participation that older adults and their health care professionals view as critically important, and to overcome barriers to their quality measurement. In particular, despite the inherent methodological challenges involved in economic evaluation of health promotion programs [41], it is vital that cost-effectiveness data are provided in order to help justify future investment by policy makers in PA interventions for older adults.

We envision that the inventory of outcome domains and subdomains generated with this review will have multiple applications. First, we expect the review will serve as essential background information (an ‘informative brief’) toward the development of a COS for PA RCTs with older adults [28, 42]. Indeed, one third of published COS are preceded by a literature review to identify potentially relevant outcomes [23]. Thus, our review findings will be used as an evidence summary during future patient and health care provider engagement as well as consensus generation activities that lead to development of a COS. For instance, we will seek to compare patient and health care professional priorities for outcome domains with the inventory of outcome domains/subdomains generated by this review to identify both areas of overlap and difference. Second, as development work on a COS proceeds, the outputs of this review will be useful immediately to inform outcome selection for RCTs of PA for older adults and to guide reporting in systematic reviews and meta-analyses that synthesize RCTs of PA for older adults.

This study had certain limitations. First, our inclusion criteria were not designed to identify a complete and exhaustive set of older adult PA RCTs; rather, we sought to identify enough representative trials to achieve sufficient understanding of the concepts necessary to address the study objectives. We recognize, for example, that by including only RCTs published in journals with a top-10 ranking, we may have missed outcome domains and subdomains found in articles published in lower ranking journals. Second, in this rapid review, we did not formally assess study quality of the 67 included articles with a published checklist or tool, as would be required for a systematic review about intervention effectiveness. Formal quality assessment was not necessary to achieve the objectives of this review, as our purpose was not to assess effectiveness of PA interventions, but rather to create an inventory of outcome domains and associated subdomains that have been used in past PA RCTs. Moreover, we attempted to locate high-quality studies at the outset through searching exclusively for RCTs published in top-10 journals in the four most relevant disciplines. Third, we did not consider factors that may affect selection of outcome measurement instruments such as psychometric properties, feasibility, or mode of assessment, nor did we explore differences in outcome selection based on trial characteristics that may influence adoption of outcome measurement instruments. This was in keeping with the purpose of this review to generate an inventory of outcomes used in previous RCTs of PA for older adults to inform the selection of outcome domains (‘what’ to measure) for inclusion in a future COS. The selection of outcome instruments (‘how’ to measure) is distinct from, and typically comes after, the selection of outcome domains. Fourth, the results of this rapid review were influenced by outcome selection in the LIFE Study and the LIFE Pilot Study, as 21% of included articles reported results from these trials, reflecting the profound impact of these trials in the field of aging and PA. Nonetheless, a wide range of outcome domains were identified in these articles, including musculoskeletal and connective tissue, general, injury and poisoning, nervous system, psychiatric, vascular, physical functioning, social functioning, emotional functioning/wellbeing, cognitive functioning, and economic, diminishing the probability of overweighting certain domains from these two trials. Outcome selection in LIFE Study and the LIFE Pilot Study was governed predominantly by trial investigators [43, 44]; moreover, the inventory of outcome domains reported in this review most likely reflect the priorities of clinical trial investigators. Since outcomes selected for a COS must be meaningful to researchers, patients, and health care professionals, there is a distinct opportunity to advance the field by documenting and incorporating outcome preferences of older adults and their health care professionals in a COS [7, 45].

## Conclusions

In conclusion this review provides an inventory of outcome domains and subdomains used in recent, high-quality RCTs of PA for older adults. There is strong potential to integrate the results of this review with the top research priorities of older adults and their health care professionals into a future COS for PA RCTs for older adults.

## Supplementary Information

**Additional file 1.** Search strategy.

**Additional file 2.** Complete list of core areas and outcome domains from the COMET taxonomy and custom outcome subdomains derived by the authors.

**Additional file 3.** Applying the COMET taxonomy to some outcomes required discussion among the researchers; decisions about outcome classification are detailed within.

**Additional file 4.** List of included articles in the rapid review (*n*=67), organized alphabetically.

## Data Availability

The dataset generated and analyzed during the current study are available from the corresponding author on reasonable request.

## Abbreviations

COMET: Core Outcome Measures in Effectiveness Trials
COS: Core Outcome Set
LIFE: Lifestyle Interventions and Independence for Elders
PA: Physical Activity
PRISMA: Preferred Reporting Items for Systematic Reviews and Meta-Analyses
PRISMA-ScR: Preferred Reporting Items for Systematic Reviews and Meta-Analyses extension for scoping review
RCT: Randomized Controlled Trial
