# Supplementary material for: Outcome domains measured in randomized controlled trials of physical activity for older adults: a rapid review
Source: Int J Behav Nutr Phys Act. 2023 Mar 24;20:34. doi: 10.1186/s12966-023-01431-3 (PMC10039503; doi:10.1186/s12966-023-01431-3)
Supplement: Supplementary file 1 — Additional file 1. Search strategy. [file 12966_2023_1431_MOESM1_ESM.docx]

Outcome domains measured in randomized controlled trials of physical activity for older adults: A rapid review

Additional file 1

Search Strategy

We conducted our search using Ovid MEDLINE.

Ovid help was available at: https://ospguides.ovid.com/OSPguides/medline.htm

**most recent search on February 17, 2021 by DCM

**start by searching for relevant journal titles

**search for top 10 journals in category = MEDICINE, GENERAL & INTERNAL (n=165 journals total)

(new england journal of medicine or lancet or jama or nature reviews disease primers or british medical journal or annals of internal medicine or jama internal medicine or plos medicine public library of science or "journal of cachexia sarcopenia and muscle" or Cochrane database of systematic reviews).jn

[records=451995]

**search for top 10 journals in category = GERIATRICS & GERONTOLOGY (n=51 journals total)

(ageing research reviews or "journal of cachexia sarcopenia and muscle" or aging cell or "aging and disease" or journals of gerontology series a biological sciences & medical sciences or age & ageing or aging or journal of the american medical directors association or frontiers in aging neuroscience or neurobiology of aging).jn

[records=34333]

**search for top 10 journals in category = SPORTS SCIENCE (n=85 journals total)

(british journal of sports medicine or sports medicine or american journal of sports medicine or exercise immunology review or journal of sport & health science or journal of the international society of sports nutrition or exercise & sport sciences reviews or arthroscopy or medicine & science in sports & exercise or international journal of sport nutrition & exercise metabolism).jn [records=41189]

**search for top 10 journals in category = REHABILITATION (n=68 journals total)

(journal of physiotherapy or neurorehabilitation & neural repair or journal of orthopaedic & sports physical therapy or annals of physical & rehabilitation medicine or journal of neuroengineering & rehabilitation or journal of geriatric physical therapy or ieee transactions on neural systems & rehabilitation engineering or physical therapy or archives of physical medicine & rehabilitation or journal of neurologic physical therapy).jn

[records=32396]

**for journal titles, use “&” instead of “and”

**combine four categories of journals

**A

1 or 2 or 3 or 4

[records=559078]

**search for concept = physical activity with MESH terms. MESH terms do not have to be major terms (e.g., exp Exercise Therapy/ rather than exp *Exercise Therapy/).

exp Exercise Therapy/ [records=52946]

exp Exercise/ [records=203577]

**add that ‘physical activity’ or ‘exercise’ could also be title, abstract, or keywords. By including a few title, abstract, and key words, we will not be entirely reliant on MESH terms for the physical activity concept, and thus will pull up more recent articles that do not yet have MESH terms assigned. ‘kf’ is keyword heading word and is a bit broader than ‘kw’, which is author assigned keyword heading; use ‘kf’

physical activit*.ti,ab,kf [records=118514]

exercis*.ti,ab,kf [records=307303]

**B

(exp Exercise Therapy/ OR exp Exercise/ OR physical activit*.ti,ab,kf OR exercis*.ti,ab,kf) [records=484609]

**search for concept = older adult with MESH terms and title, abstract, or keywords

exp Aging/ [records=249255]

exp Healthy Aging/ [records=1122]

exp Aged/ [records=3199583]

(aging or ageing or age-related or older population or older adult* or older people* or older person* or older age* or older man or older men or older male* or older woman or older women or older female* or elderl* or senior* or senior citizen*).ti,ab,kf

[records=693870]

**C

exp Aging/ OR exp Healthy Aging/ OR exp Aged/ OR (aging or ageing or age-related or old age* or geriatri* or older population or older adult* or older people* or older person* or older age* or older man or older men or older male* or older woman or older women or older female* or elderl* or senior* or senior citizen*).ti,ab,kf [records=3629325]

**Overall search strategy: search for journals AND physical activity concept AND apply filters for publication year, RCT, age group = Elderly

**Journals (A) AND physical activity concept (B) AND older adult concept (C)

A&B&C

[records=8319]

**limit to randomized controlled trial

[records=1544]

**limit to year=2015-present

[records=548]

**limit to English language

[records=548]

**limit to Humans

[records=548]
