# Supplementary material for: Outcome domains measured in randomized controlled trials of physical activity for older adults: a rapid review
Source: Int J Behav Nutr Phys Act. 2023 Mar 24;20:34. doi: 10.1186/s12966-023-01431-3 (PMC10039503; doi:10.1186/s12966-023-01431-3)
Supplement: Supplementary file 2 — Additional file 2. Complete list of core areas and outcome domains from the COMET taxonomy and custom outcome subdomains derived by the authors. [file 12966_2023_1431_MOESM2_ESM.docx]

Outcome domains measured in randomized controlled trials of physical activity for older adults:
A rapid review

Additional file 2

Complete list of core areas and outcome domains from the COMET taxonomy [1] and custom outcome subdomains derived by the authors that were used to classify outcomes. Areas, domains, and subdomains that were identified in the n=67 articles in this review (✓) are presented in Table 3.

| **Core Area** | **Outcome Domain** | **Outcome Subdomain** | **Identified** |
| --- | --- | --- | --- |
| **Death** | 1. Mortality/survival |  |  |
| **Physiological/ Clinical** | 2. Blood and lymphatic system outcomes |  |  |
|  | 3. Cardiac outcomes |  | ✓ |
|  | 4. Congenital, familial and genetic outcomes |  |  |
|  | 5. Endocrine outcomes |  | ✓ |
|  | 6. Ear and labyrinth outcomes |  |  |
|  | 7. Eye outcomes |  | ✓ |
|  | 8. Gastrointestinal outcomes |  |  |
|  | 9. General outcomes | 9a. Fall-related | ✓ |
|  |  | 9b. Body composition | ✓ |
|  |  | 9c. Other | ✓ |
|  | 10. Hepatobiliary outcomes |  |  |
|  | 11. Immune system outcomes |  | ✓ |
|  | 12. Infection and infestation outcomes |  | ✓ |
|  | 13. Injury and poisoning outcomes |  | ✓ |
|  | 14. Metabolism and nutrition outcomes |  | ✓ |
|  | 15. Musculoskeletal and connective tissue outcomes | 15a. Muscle performance | ✓ |
|  |  | 15b. Bone | ✓ |
|  |  | 15c. Body composition | ✓ |
|  |  | 15d. Other | ✓ |
|  | 16. Outcomes relating to neoplasms: benign, malignant and unspecified (including cysts and polyps) |  |  |
|  | 17. Nervous system outcomes |  | ✓ |
|  | 18. Pregnancy, puerperium and perinatal outcomes |  |  |
|  | 19. Renal and urinary outcomes |  |  |
|  | 20. Reproductive system and breast outcomes |  |  |
|  | 21. Psychiatric outcomes |  | ✓ |
|  | 22. Respiratory, thoracic and mediastinal outcomes |  | ✓ |
|  | 23. Skin and subcutaneous tissue outcomes |  |  |
|  | 24. Vascular outcomes |  | ✓ |
| **Life Impact** | 25. Physical functioning | 25a. Mobility | ✓ |
|  |  | 25b. Fall-related | ✓ |
|  |  | 25c. Lifestyle | ✓ |
|  |  | 25d. Balance | ✓ |
|  |  | 25e. Quality of Life | ✓ |
|  |  | 25f. Other | ✓ |
|  | 26. Social functioning |  | ✓ |
|  | 27. Role functioning |  | ✓ |
|  | 28. Emotional functioning/wellbeing | 28a. Fall-related | ✓ |
|  |  | 28b. Quality of life | ✓ |
|  | 29. Cognitive functioning | 29a. Multiple cognitive functions | ✓ |
|  |  | 29b. Processing speed & executive functions | ✓ |
|  |  | 29c. Other | ✓ |
|  | 30. Global quality of life |  |  |
|  | 31. Perceived health status |  |  |
|  | 32. Delivery of care |  | ✓ |
|  | 33. Personal circumstances |  |  |
| **Resource Use** | 34. Economic |  | ✓ |
|  | 35. Hospital |  |  |
|  | 36. Need for further intervention |  |  |
|  | 37. Societal/carer burden |  |  |
| **Adverse Events** | 38. Adverse events/effects |  | ✓ |

**Reference**

1. Dodd S, Clarke M, Becker L, Mavergames C, Fish R, Williamson PR. A taxonomy has been developed for outcomes in medical research to help improve knowledge discovery. J. Clin. Epidemiol. United States; 2018;96:84–92.
