# Supplementary material for: Outcome domains measured in randomized controlled trials of physical activity for older adults: a rapid review
Source: Int J Behav Nutr Phys Act. 2023 Mar 24;20:34. doi: 10.1186/s12966-023-01431-3 (PMC10039503; doi:10.1186/s12966-023-01431-3)
Supplement: Supplementary file 3 — Additional file 3. Applying the COMET taxonomy to some outcomes required discussion among the researchers; decisions about outcome classification are detailed within. [file 12966_2023_1431_MOESM3_ESM.docx]

Outcome domains measured in randomized controlled trials of physical activity for older adults: A rapid review

Additional file 3

Applying the COMET taxonomy [1] to some outcomes required discussion among the researchers; decisions about outcome classification are detailed here:

Fall risk assessments (e.g., Physiological Profile Assessment) were classified as ‘general outcomes’ (under core area = physiological/clinical), as such assessments often probe whole body risk.

Frailty was classified as ‘general outcomes’ (under core area = physiological/clinical) because frailty is a clinical condition affecting the whole body and cannot be attributed to a certain body system [2].

Measures of muscle strength (e.g., lower extremity, upper extremity, hand grip), muscle power, muscle fatigue, and muscle torque were classified as ‘musculoskeletal & connective tissue outcomes’ (under core area = physiological/clinical). These tests are typically direct measures of physiological function more so than daily physical function.

Measures of balance (e.g., postural sway, balance scales and tests) were classified as ‘general outcomes’ (under core area = physiological/clinical), as these tests are typically direct measures of physiological function, involving multiple body systems, more so than functional tests [2].

Functional tests (e.g., SPPB, TUG, chair stands, step tests, walking/gait tests) were classified as ‘physical functioning outcomes’ (under core area = life impact).

Dietary measures (e.g., food frequency questionnaires) were classified as ‘physical functioning outcomes’ (under core area = life impact), per personal communication and recommendation from Susana Dodd.

Vitamin D (e.g., 25(OH)D) was classified as ‘metabolism and nutrition’ (under core area = physiological/clinical).

Measures of confidence, efficacy, and fear (e.g., falls efficacy, balance confidence) were classified as ‘emotional functioning/wellbeing’ (under core area = life impact).

Physical activity and sedentary behaviour were classified as ‘physical functioning outcomes’ (under core area = life impact) because they are examples of health behaviours.

Also consistent with guidance on how to apply the COMET taxonomy [1], and following discussion among the researchers, some outcomes were classified under multiple domains.

Falls were classified as ‘general outcomes’ (under core area = physiological/clinical) and as ‘physical functioning’ (under core area = life impact). Falls are a clinically relevant health event, which may be prevented by physical activity, and may be measured in physical activity RCTs as an index of impact on physical functioning. We considered but chose not to classify falls as ‘injury & poisoning outcomes’ (under core area = physiological/clinical) because not all falls result in injury.

Fractures were classified as ‘injury & poisoning outcomes’ (under core area = physiological/clinical) and as musculoskeletal & connective tissue outcomes (also under core area = physiological/clinical) because fractures are a musculoskeletal injury.

Blood pressure measures (at all locations in the body) were classified as ‘cardiac outcomes’ and ‘vascular outcomes’ (both under core area = physiological/clinical).

**Abbreviations**COMET: Core Outcome Measures in Effectiveness Trials; RCT: Randomized Controlled Trial; SPPB: Short Physical Performance Battery; TUG: Timed Up and Go

**References**

1. Dodd S, Clarke M, Becker L, Mavergames C, Fish R, Williamson PR. A taxonomy has been developed for outcomes in medical research to help improve knowledge discovery. J. Clin. Epidemiol. United States; 2018;96:84–92.

2. Baldwin CE, Phillips AC, Edney SM, Lewis LK. Core domains for research on hospital inactivity in acutely ill older adults: A delphi consensus study. Arch. Phys. Med. Rehabil. United States; 2021;102:664–74.
