# Supplementary material for: Outcome domains measured in randomized controlled trials of physical activity for older adults: a rapid review
Source: Int J Behav Nutr Phys Act. 2023 Mar 24;20:34. doi: 10.1186/s12966-023-01431-3 (PMC10039503; doi:10.1186/s12966-023-01431-3)
Supplement: Supplementary file 4 — Additional file 4. List of included articles in the rapid review (n=67), organized alphabetically. [file 12966_2023_1431_MOESM4_ESM.docx]

Outcome domains measured in randomized controlled trials of physical activity for older adults: A rapid review

Additional file 4

List of included articles in the rapid review (n=67), organized alphabetically

Allen JD, Vanbruggen MD, Johannsen NM, Robbins JL, Credeur DP, Pieper CF, et al. PRIME: A Novel Low-Mass, High-Repetition Approach to Improve Function in Older Adults. Medicine and science in sports and exercise. 2018;50:1005–14. doi: 10.1249/MSS.0000000000001518

Alqahtani BA, Sparto PJ, Whitney SL, Greenspan SL, Perera S, VanSwearingen J, et al. Effect of Community-Based Group Exercise Interventions on Standing Balance and Strength in Independent Living Older Adults. Journal of geriatric physical therapy. 2019;42:E7–15. doi: 10.1519/JPT.0000000000000221

Arkkukangas M, Soderlund A, Eriksson S, Johansson A-C. Fall Preventive Exercise With or Without Behavior Change Support for Community-Dwelling Older Adults: A Randomized Controlled Trial With Short-Term Follow-up. Journal of geriatric physical therapy (2001). 2019;42:9–17. doi: 10.1519/JPT.0000000000000129

Bates A, Furber S, Tiedemann A, Ginn K, van den Dolder P, Howard K, et al. Trial Protocol: Home-based exercise programs to prevent falls and upper limb dysfunction among community-dwelling older people: study protocol for the BEST (Balance Exercise Strength Training) at Home randomised, controlled trial. Journal of physiotherapy. 2018;64:121. doi: 10.1016/j.jphys.2017.10.001

Bellumori M, Uygur M, Knight CA. High-Speed Cycling Intervention Improves Rate-Dependent Mobility in Older Adults. Medicine and science in sports and exercise. 2017;49:106–14. doi: 10.1249/MSS.0000000000001069

Bischoff-Ferrari HA, Vellas B, Rizzoli R, Kressig RW, da Silva JAP, Blauth M, et al. Effect of Vitamin D Supplementation, Omega-3 Fatty Acid Supplementation, or a Strength-Training Exercise Program on Clinical Outcomes in Older Adults: The DO-HEALTH Randomized Clinical Trial. JAMA. 2020;324:1855–68. doi: 10.1001/jama.2020.16909

Brach JS, Lowry K, Perera S, Hornyak V, Wert D, Studenski SA, et al. Improving motor control in walking: a randomized clinical trial in older adults with subclinical walking difficulty. Archives of physical medicine and rehabilitation. 2015;96:388–94. doi: 10.1016/j.apmr.2014.10.018

Buford TW, Miller ME, Church TS, Gill TM, Henderson R, Hsu F-C, et al. Antihypertensive Use and the Effect of a Physical Activity Intervention in the Prevention of Major Mobility Disability Among Older Adults: The LIFE Study. The journals of gerontology Series A, Biological sciences and medical sciences. 2016;71:974–81. doi: 10.1093/gerona/glv222

Buskard ANL, Jacobs KA, Eltoukhy MM, Strand KL, Villanueva L, Desai PP, et al. Optimal Approach to Load Progressions during Strength Training in Older Adults. Medicine and science in sports and exercise. 2019;51:2224–33. doi: 10.1249/MSS.0000000000002038

Cao Dinh H, Njemini R, Onyema OO, Beyer I, Liberman K, De Dobbeleer L, et al. Strength Endurance Training but Not Intensive Strength Training Reduces Senescence-Prone T Cells in Peripheral Blood in Community-Dwelling Elderly Women. The journals of gerontology Series A, Biological sciences and medical sciences. 2019;74:1870–8. doi: 10.1093/gerona/gly229

Cesari M, Vellas B, Hsu F-C, Newman AB, Doss H, King AC, et al. A physical activity intervention to treat the frailty syndrome in older persons-results from the LIFE-P study. The journals of gerontology Series A, Biological sciences and medical sciences. 2015;70:216–22. doi: 10.1093/gerona/glu099

Day L, Hill KD, Stathakis VZ, Flicker L, Segal L, Cicuttini F, et al. Impact of tai-chi on falls among preclinically disabled older people. A randomized controlled trial. Journal of the American Medical Directors Association. 2015;16:420–6. doi: 10.1016/j.jamda.2015.01.089

Duckham RL, Masud T, Taylor R, Kendrick D, Carpenter H, Iliffe S, et al. Randomised controlled trial of the effectiveness of community group and home-based falls prevention exercise programmes on bone health in older people: the ProAct65+ bone study. Age and ageing. 2015;44:573–9. doi: 10.1093/ageing/afv055

Duff WRD, Chilibeck PD, Candow DG, Gordon JJ, Mason RS, Taylor-Gjevre R, et al. Effects of Ibuprofen and Resistance Training on Bone and Muscle: A Randomized Controlled Trial in Older Women. Medicine and science in sports and exercise. 2017;49:633–40. doi: 10.1249/MSS.0000000000001172

Englund DA, Price LL, Grosicki GJ, Iwai M, Kashiwa M, Liu C, et al. Progressive Resistance Training Improves Torque Capacity and Strength in Mobility-Limited Older Adults. The journals of gerontology Series A, Biological sciences and medical sciences. 2019;74:1316–21. doi: 10.1093/gerona/gly199

Espejo-Antunez L, Perez-Marmol JM, Cardero-Duran M de LA, Toledo-Marhuenda JV, Albornoz-Cabello M. The Effect of Proprioceptive Exercises on Balance and Physical Function in Institutionalized Older Adults: A Randomized Controlled Trial. Archives of physical medicine and rehabilitation. 2020;101:1780–8. doi: 10.1016/j.apmr.2020.06.010

Espeland MA, Lipska K, Miller ME, Rushing J, Cohen RA, Verghese J, et al. Effects of Physical Activity Intervention on Physical and Cognitive Function in Sedentary Adults With and Without Diabetes. The journals of gerontology Series A, Biological sciences and medical sciences. 2017;72:861–6. doi: 10.1093/gerona/glw179

Fanning J, Rejeski WJ, Chen S-H, Nicklas BJ, Walkup MP, Axtell RS, et al. A Case for Promoting Movement Medicine: Preventing Disability in the LIFE Randomized Controlled Trial. The journals of gerontology Series A, Biological sciences and medical sciences. 2019;74:1821–7. doi: 10.1093/gerona/glz050

Forti LN, Van Roie E, Njemini R, Coudyzer W, Beyer I, Delecluse C, et al. Load-Specific Inflammation Mediating Effects of Resistance Training in Older Persons. Journal of the American Medical Directors Association. 2016;17:547–52. doi: 10.1016/j.jamda.2016.02.010

Franco MR, Sherrington C, Tiedemann A, Pereira LS, Perracini MR, Faria CSG, et al. Effect of Senior Dance (DanSE) on Fall Risk Factors in Older Adults: A Randomized Controlled Trial. Physical therapy. 2020;100:600–8. doi: 10.1093/ptj/pzz187

Fu AS, Gao KL, Tung AK, Tsang WW, Kwan MM. Effectiveness of Exergaming Training in Reducing Risk and Incidence of Falls in Frail Older Adults With a History of Falls. Archives of physical medicine and rehabilitation. 2015;96:2096–102. doi: 10.1016/j.apmr.2015.08.427

Gallo E, Stelmach M, Frigeri F, Ahn D-H. Determining Whether a Dosage-Specific and Individualized Home Exercise Program With Consults Reduces Fall Risk and Falls in Community-Dwelling Older Adults With Difficulty Walking: A Randomized Control Trial. Journal of geriatric physical therapy (2001). 2018;41:161–72. Doi: 10.1519/JPT.0000000000000114

Gill TM, Guralnik JM, Pahor M, Church T, Fielding RA, King AC, et al. Effect of Structured Physical Activity on Overall Burden and Transitions Between States of Major Mobility Disability in Older Persons: Secondary Analysis of a Randomized Trial. Annals of internal medicine. 2016;165:833–40. doi: 10.7326/M16-0529

Glenn JM, Gray M, Binns A. The effects of loaded and unloaded high-velocity resistance training on functional fitness among community-dwelling older adults. Age and ageing. 2015;44:926–31. doi: 10.1093/ageing/afv081

Gordon N, Abbiss CR, Maiorana AJ, James AP, Clark K, Marston KJ, et al. High-Intensity Single-Leg Cycling Improves Cardiovascular Disease Risk Factor Profile. Medicine and science in sports and exercise. 2019;51:2234–42. doi: 10.1249/MSS.0000000000002053

Gothe NP, McAuley E. Yoga Is as Good as Stretching-Strengthening Exercises in Improving Functional Fitness Outcomes: Results From a Randomized Controlled Trial. The journals of gerontology Series A, Biological sciences and medical sciences. 2016;71:406–11. doi: 10.1093/gerona/glv127

Groessl EJ, Kaplan RM, Castro Sweet CM, Church T, Espeland MA, Gill TM, et al. Cost-effectiveness of the LIFE Physical Activity Intervention for Older Adults at Increased Risk for Mobility Disability. The journals of gerontology Series A, Biological sciences and medical sciences. 2016;71:656–62. doi: 10.1093/gerona/glw001

Haynes A, Naylor LH, Carter HH, Spence AL, Robey E, Cox KL, et al. Land-walking vs. water-walking interventions in older adults: Effects on aerobic fitness. Journal of sport and health science. 2020;9:274–82. doi: 10.1016/j.jshs.2019.11.005

Henderson RM, Lovato L, Miller ME, Fielding RA, Church TS, Newman AB, et al. Effect of Statin Use on Mobility Disability and its Prevention in At-risk Older Adults: The LIFE Study. The journals of gerontology Series A, Biological sciences and medical sciences. 2016;71:1519–24. doi: 10.1093/gerona/glw057

Henderson RM, Miller ME, Fielding RA, Gill TM, Glynn NW, Guralnik JM, et al. Maintenance of Physical Function 1 Year After Exercise Intervention in At-Risk Older Adults: Follow-up From the LIFE Study. The journals of gerontology Series A, Biological sciences and medical sciences. 2018;73:688–94. doi: 10.1093/gerona/glx231

Hewitt J, Goodall S, Clemson L, Henwood T, Refshauge K. Progressive Resistance and Balance Training for Falls Prevention in Long-Term Residential Aged Care: A Cluster Randomized Trial of the Sunbeam Program. Journal of the American Medical Directors Association. 2018;19:361–9. doi: 10.1016/j.jamda.2017.12.014

Hirase T, Inokuchi S, Matsusaka N, Okita M. Effects of a balance training program using a foam rubber pad in community-based older adults: a randomized controlled trial. Journal of geriatric physical therapy (2001). 2015;38:62–70. doi: 10.1519/JPT.0000000000000023

Karinkanta S, Kannus P, Uusi-Rasi K, Heinonen A, Sievanen H. Combined resistance and balance-jumping exercise reduces older women’s injurious falls and fractures: 5-year follow-up study. Age and ageing. 2015;44:784–9. doi: 10.1093/ageing/afv064

Kim H-K, Hwang C-L, Yoo J-K, Hwang M-H, Handberg EM, Petersen JW, et al. All-Extremity Exercise Training Improves Arterial Stiffness in Older Adults. Medicine and science in sports and exercise. 2017;49:1404–11. doi: 10.1249/MSS.0000000000001229

Kitazawa K, Showa S, Hiraoka A, Fushiki Y, Sakauchi H, Mori M. Effect of a dual-task net-step exercise on cognitive and gait function in older adults. Journal of geriatric physical therapy (2001). 2015;38:133–40. doi: 10.1519/JPT.0000000000000029

Kwok BC, Pua YH. Effects of WiiActive exercises on fear of falling and functional outcomes in community-dwelling older adults: a randomised control trial. Age and ageing. 2016;45:621–7. doi: 10.1093/ageing/afw108

Lamb SE, Bruce J, Hossain A, Ji C, Longo R, Lall R, et al. Screening and Intervention to Prevent Falls and Fractures in Older People. The New England journal of medicine. 2020;383:1848–59. doi: 10.1056/NEJMoa2001500

Layne AS, Hsu F-C, Blair SN, Chen S-H, Dungan J, Fielding RA, et al. Predictors of Change in Physical Function in Older Adults in Response to Long-Term, Structured Physical Activity: The LIFE Study. Archives of physical medicine and rehabilitation. 2017;98:11-24.e3. doi: 10.1016/j.apmr.2016.07.019

Li F, Harmer P, Chou L-S. Dual-Task Walking Capacity Mediates Tai Ji Quan Impact on Physical and Cognitive Function. Medicine and science in sports and exercise. 2019;51:2318–24. doi: 10.1249/MSS.0000000000002051

Li F, Harmer P, Fitzgerald K, Eckstrom E, Akers L, Chou L-S, et al. Effectiveness of a Therapeutic Tai Ji Quan Intervention vs a Multimodal Exercise Intervention to Prevent Falls Among Older Adults at High Risk of Falling: A Randomized Clinical Trial. JAMA internal medicine. 2018;178:1301–10. doi: 10.1001/jamainternmed.2018.3915

Liew LK, Tan MP, Tan PJ, Mat S, Majid LA, Hill KD, et al. The Modified Otago Exercises Prevent Grip Strength Deterioration Among Older Fallers in the Malaysian Falls Assessment and Intervention Trial (MyFAIT). Journal of geriatric physical therapy (2001). 2019;42:123–9. doi: 10.1519/JPT.0000000000000155

Liu-Ambrose T, Davis JC, Best JR, Dian L, Madden K, Cook W, et al. Effect of a Home-Based Exercise Program on Subsequent Falls Among Community-Dwelling High-Risk Older Adults After a Fall: A Randomized Clinical Trial. JAMA. 2019;321:2092–100. doi: 10.1001/jama.2019.5795

Marengoni A, Rizzuto D, Fratiglioni L, Antikainen R, Laatikainen T, Lehtisalo J, et al. The Effect of a 2-Year Intervention Consisting of Diet, Physical Exercise, Cognitive Training, and Monitoring of Vascular Risk on Chronic Morbidity-the FINGER Randomized Controlled Trial. Journal of the American Medical Directors Association. 2018;19:355-360.e1. doi: 10.1016/j.jamda.2017.09.020

Markofski MM, Jennings K, Timmerman KL, Dickinson JM, Fry CS, Borack MS, et al. Effect of Aerobic Exercise Training and Essential Amino Acid Supplementation for 24 Weeks on Physical Function, Body Composition, and Muscle Metabolism in Healthy, Independent Older Adults: A Randomized Clinical Trial. The journals of gerontology Series A, Biological sciences and medical sciences. 2019;74:1598–604. doi: 10.1093/gerona/gly109

Mejias-Pena Y, Estebanez B, Rodriguez-Miguelez P, Fernandez-Gonzalo R, Almar M, de Paz JA, et al. Impact of resistance training on the autophagy-inflammation-apoptosis crosstalk in elderly subjects. Aging. 2017;9:408–18. doi: 10.18632/aging.101167

Merom D, Mathieu E, Cerin E, Morton RL, Simpson JM, Rissel C, et al. Social Dancing and Incidence of Falls in Older Adults: A Cluster Randomised Controlled Trial. PLoS medicine. 2016;13:e1002112. doi: 10.1371/journal.pmed.1002112

Minett MM, Binkley TL, Holm RP, Runge M, Specker BL. Feasibility and Effects on Muscle Function of an Exercise Program for Older Adults. Medicine and science in sports and exercise. 2020;52:441–8. doi: 10.1249/MSS.0000000000002152

Nadkarni NK, Perera S, Studenski SA, Rosano C, Aizenstein HJ, VanSwearingen JM. Callosal hyperintensities and gait speed gain from two types of mobility interventions in older adults. Archives of physical medicine and rehabilitation. 2015;96:1154–7. doi: 10.1016/j.apmr.2014.09.026

Pessoa MF, Brandao DC, Sa RB de, Barcelar J de M, Rocha TD de S, Souza HCM de, et al. Vibrating Platform Training Improves Respiratory Muscle Strength, Quality of Life, and Inspiratory Capacity in the Elderly Adults: A Randomized Controlled Trial. The journals of gerontology Series A, Biological sciences and medical sciences. 2017;72:683–8. doi: 10.1093/gerona/glw123

Reid KF, Laussen J, Bhatia K, Englund DA, Kirn DR, Price LL, et al. Translating the Lifestyle Interventions and Independence for Elders Clinical Trial to Older Adults in a Real-World Community-Based Setting. The journals of gerontology Series A, Biological sciences and medical sciences. 2019;74:924–8. doi: 10.1093/gerona/gly152

Reid KF, Martin KI, Doros G, Clark DJ, Hau C, Patten C, et al. Comparative effects of light or heavy resistance power training for improving lower extremity power and physical performance in mobility-limited older adults. The journals of gerontology Series A, Biological sciences and medical sciences. 2015;70:374–80. doi: 10.1093/gerona/glu156

Ribeiro AS, Deminice R, Schoenfeld BJ, Tomeleri CM, Padilha CS, Venturini D, et al. Effect of Resistance Training Systems on Oxidative Stress in Older Women. International journal of sport nutrition and exercise metabolism. 2017;27:439–47. doi: 10.1123/ijsnem.2016-0322

Shaaban CE, Aizenstein HJ, Jorgensen DR, Mahbubani RLM, Meckes NA, Erickson KI, et al. Physical Activity and Cerebral Small Vein Integrity in Older Adults. Medicine and science in sports and exercise. 2019;51:1684–91. doi: 10.1249/MSS.0000000000001952

Shimada H, Ishii K, Makizako H, Ishiwata K, Oda K, Suzukawa M. Effects of exercise on brain activity during walking in older adults: a randomized controlled trial. Journal of neuroengineering and rehabilitation. 2017;14:50. doi: 10.1186/s12984-017-0263-9

Sink KM, Espeland MA, Castro CM, Church T, Cohen R, Dodson JA, et al. Effect of a 24-Month Physical Activity Intervention vs Health Education on Cognitive Outcomes in Sedentary Older Adults: The LIFE Randomized Trial. JAMA. 2015;314:781. doi: 10.1001/jama.2015.9617

Son N-K, Ryu YU, Jeong H-W, Jang Y-H, Kim H-D. Comparison of 2 Different Exercise Approaches: Tai Chi Versus Otago, in Community-Dwelling Older Women. Journal of geriatric physical therapy (2001). 2016;39:51–7. doi: 10.1519/JPT.0000000000000042

Strandberg E, Ponsot E, Piehl-Aulin K, Falk G, Kadi F. Resistance Training Alone or Combined With N-3 PUFA-Rich Diet in Older Women: Effects on Muscle Fiber Hypertrophy. The journals of gerontology Series A, Biological sciences and medical sciences. 2019;74:489–94. doi: 10.1093/gerona/gly130

Ten Brinke LF, Best JR, Chan JLC, Ghag C, Erickson KI, Handy TC, et al. The Effects of Computerized Cognitive Training With and Without Physical Exercise on Cognitive Function in Older Adults: An 8-Week Randomized Controlled Trial. The journals of gerontology Series A, Biological sciences and medical sciences. 2020;75:755–63. doi: 10.1093/gerona/glz115

Trombetti A, Hars M, Hsu F-C, Reid KF, Church TS, Gill TM, et al. Effect of Physical Activity on Frailty: Secondary Analysis of a Randomized Controlled Trial. Annals of internal medicine. 2018;168:309–16. doi: 10.7326/M16-2011

Urzi F, Marusic U, Licen S, Buzan E. Effects of Elastic Resistance Training on Functional Performance and Myokines in Older Women-A Randomized Controlled Trial. Journal of the American Medical Directors Association. 2019;20:830-834.e2. doi: 10.1016/j.jamda.2019.01.151

Uusi-Rasi K, Patil R, Karinkanta S, Kannus P, Tokola K, Lamberg-Allardt C, et al. Exercise and vitamin D in fall prevention among older women: a randomized clinical trial. JAMA internal medicine. 2015;175:703–11. doi: 10.1001/jamainternmed.2015.0225

Uusi-Rasi K, Patil R, Karinkanta S, Kannus P, Tokola K, Lamberg-Allardt C, et al. A 2-Year Follow-Up After a 2-Year RCT with Vitamin D and Exercise: Effects on Falls, Injurious Falls and Physical Functioning Among Older Women. The Journals of Gerontology: Series A. 2017;72:1239–45. doi: 10.1093/gerona/glx044

Voss MW, Weng TB, Narayana-Kumanan K, Cole RC, Wharff C, Reist L, et al. Acute Exercise Effects Predict Training Change in Cognition and Connectivity. Medicine and science in sports and exercise. 2020;52:131–40. doi: 10.1249/MSS.0000000000002115

Voukelatos A, Merom D, Sherrington C, Rissel C, Cumming RG, Lord SR. The impact of a home-based walking programme on falls in older people: the Easy Steps randomised controlled trial. Age and ageing. 2015;44:377–83. doi: 10.1093/ageing/afu186

Wanigatunga AA, Tudor-Locke C, Axtell RS, Glynn NW, King AC, McDermott MM, et al. Effects of a Long-Term Physical Activity Program on Activity Patterns in Older Adults. Medicine and science in sports and exercise. 2017;49:2167–75. doi: 10.1249/MSS.0000000000001340

Wojcicki TR, Fanning J, Awick EA, Olson EA, Motl RW, McAuley E. Maintenance Effects of a DVD-Delivered Exercise Intervention on Physical Function in Older Adults. The journals of gerontology Series A, Biological sciences and medical sciences. 2015;70:785–9. doi: 10.1093/gerona/glu188

Yamada M, Nishiguchi S, Fukutani N, Aoyama T, Arai H. Mail-Based Intervention for Sarcopenia Prevention Increased Anabolic Hormone and Skeletal Muscle Mass in Community-Dwelling Japanese Older Adults: The INE (Intervention by Nutrition and Exercise) Study. Journal of the American Medical Directors Association. 2015;16:654–60. doi: 10.1016/j.jamda.2015.02.017
